# Supplementary figures and images for: Excess Heritability Contribution of Alcohol Consumption Variants in the “Missing Heritability” of Type 2 Diabetes Mellitus
Source: Int J Mol Sci. 2021 Nov 15;22(22):12318. doi: 10.3390/ijms222212318 (PMC8623960; doi:10.3390/ijms222212318)

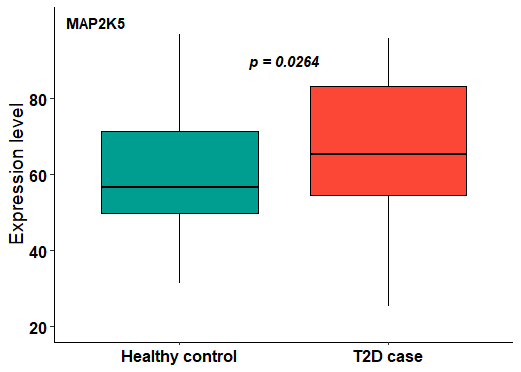

Supplement: Supplementary file 1 [file ijms-22-12318-s001.zip › MAP2K5.tiff]

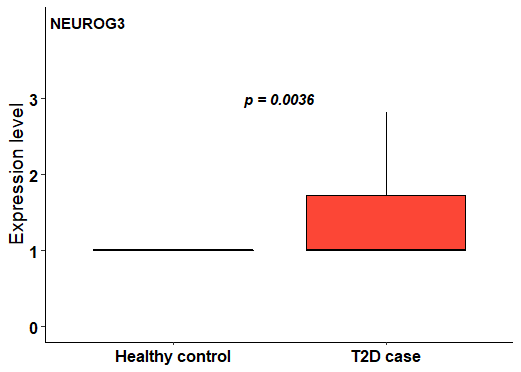

Supplement: Supplementary file 1 [file ijms-22-12318-s001.zip › NEUROG3.tiff]

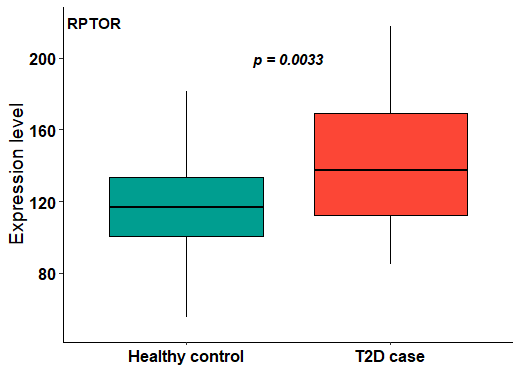

Supplement: Supplementary file 1 [file ijms-22-12318-s001.zip › RPTOR.tiff]

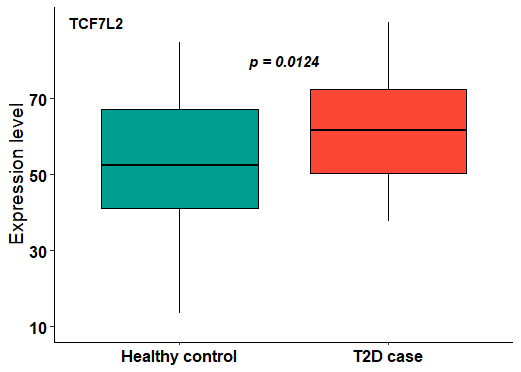

Supplement: Supplementary file 1 [file ijms-22-12318-s001.zip › TCF7L2.tiff]
